# Radiation-Induced Changes of microRNA Expression Profiles in Radiosensitive and Radioresistant Leukemia Cell Lines with Different Levels of Chromosome Abnormalities

**DOI:** 10.3390/cancers9100136

**Published:** 2017-10-13

**Authors:** Daria Liamina, Wladimir Sibirnyj, Anna Khokhlova, Viacheslav Saenko, Eugenia Rastorgueva, Aleksandr Fomin, Yury Saenko

**Affiliations:** 1 Laboratory of Molecular and Cell Biology, S.P. Kapitsa Research Institute of Technology, Ulyanovsk State University, 42 Lva Tolstogo St., Ulyanovsk 432017, Russia; daryaantonovna@yandex.ru (D.L.); avhohlova@gmail.com (A.K.); saenkoslava@mail.ru (V.S.); 2 Department of Bioenergetics and Food Analysis, Faculty of Biology and Agriculture, University of Rzeszow, Ćwiklińskiej St., 35-601 Rzeszów, Poland; vladimir_sibirnyi@yahoo.com; 3 Department of General and Clinical Pharmacology and Microbiology, Faculty of Medicine, Ulyanovsk State University, 42 Lva Tolstogo St., Ulyanovsk 432017, Russia; rastorgueva.e.v@yandex.ru; 4 S.P. Kapitsa Research Institute of Technology, Ulyanovsk State University, 42 Lva Tolstogo St., Ulyanovsk 432017, Russia; niti-ulsu@yandex.ru

**Keywords:** radioresistance, radiosensitivity, microRNA, signaling pathway, gene expression

## Abstract

In our study, we estimate an effect from chromosome aberrations and genome mutations on changes in microRNA expression profiles in cancer cell lines demonstrating different radiosensitivity. Here, cell viability and microRNA spectrum have been estimated 1, 4, and 24 h after irradiation. MiSeq high-throughput sequencing system (Illumina, San Diego, CA, USA) is employed to perform microRNA spectrum estimation. In the K562 cell line, the number of expressed microRNAs in chromosomes demonstrates a more pronounced variation. An analysis of microRNA effects on signaling pathway activity demonstrates differences in post-transcriptional regulation of the expression of genes included into 40 signaling pathways. In the K562 cell line, microRNA dynamics analyzed for their dependence on chromosome localization show a wider scattering of microRNA expression values for a pair of chromosomes compared to the HL-60 cell line. An analysis of microRNAs expression in the K562 and HL-60 cell lines after irradiation has shown that chromosome abnormalities can affect microRNA expression changes. A study of radiation-induced changes of microRNA expression profiles in the K562 and HL-60 cell lines has revealed a dependence of microRNA expression changes on the number of chromosome aberrations and genome mutations.

## 1. Introduction

Radiation therapy is widespread for cancer treatment [1]. However, in some cases, this therapy is inefficient due to radioresistance of cancer cells. Radioresistance is a complex phenomenon associated with the ability of cancer cells to maintain clonogenic potential under exposure to therapeutic doses of ionizing radiation [2]. Ionizing radiation induces cell death, mainly due to DNA damage. In cells, the inability to repair radiation-induced DNA damage triggers programmed cell death (PCD) and the cell dies [3]. The ability to trigger PCD can be reduced in response to radiation-induced DNA damage [4]. Another radioresistance mechanism of cancer cells is their ability to abnormally activate certain signaling mechanisms, e.g., DNA damage response [5]. It is assumed that the radioresistance mechanisms of cancer cells result from genetic mutations and gene expression disturbances [6,7].

The main mechanism regulating gene expression at the posttranscriptional stage is the regulation of mRNA degradation by microRNAs, which are non-coding RNAs of 20–24 nucleotides in length. In the human genome, more than one thousand microRNAs have been discovered. Each microRNA is able to regulate thousands of microRNAs. Therefore, they play a determining role in many cellular processes including cell radiosensitivity and radioresistance. It has been shown that microRNAs regulate the expression of about 30% of protein-coding genes [8]. In recent years, it has been shown that microRNAs play a significant role in cancer pathogenesis [9]. microRNA expression changes under the influence of ionizing radiation, highlighting its contribution to cell response to ionizing radiation [10]. Some studies report a relation of certain microRNAs to cancer cells radioresistance [11,12,13]. As a result, some tens of differentially expressed microRNAs have been found in radioresistant and radiosensitive cell lines; e.g., miR-125a, miR-150, miR-425 [14], miR-324–3p [15], miR-205 [16], Lin28-let7 [17], and miR-21 [18]. These microRNAs participate in post-transcriptional regulation of such genes as WNT2B, TGF-β1, TNF, RAD52, and MDM4, which are a part of TGF-beta, WNT, Toll-like receptor, DNA-repair family, and apoptosis family signaling pathway, and play a significant role in cancer cell radioresistance [19,20,21]. Also, the presence of specific genes and signaling pathways related to cancer cell radioresistance has not been convincingly demonstrated yet. However, microRNAs specific for all radioresistant cell lines have not been detected so far.

Expression of protein-coding genes and microRNA can change in the presence of chromosome abnormalities associated with the changes in chromosome number and structure of cancer cells [22]. Chromosome abnormalities provoke changes in the DNA amount in cells; in particular, they change the number of gene copies and can affect global expression patterns [22]. Studies of different cancer cells with microarray technology have demonstrated that chromosome regions containing microRNA genes exhibit high-frequency genomic alterations [23]. Chromosome abnormalities affecting microRNA loci can change microRNA expression and affect gene expression and signaling pathway activity [24].

Thus, estimating microRNA expression and its involvement in radioresistance mechanisms, chromosome abnormalities, and genetic mutations has to be taken into account. In this study, we perform an analysis of the radiation-induced dynamics of the microRNA expression profiles of radiosensitive and radioresistant cell lines with different chromosome abnormalities.

## 2. Results

### 2.1. Cell Viability

**Figure 1:**
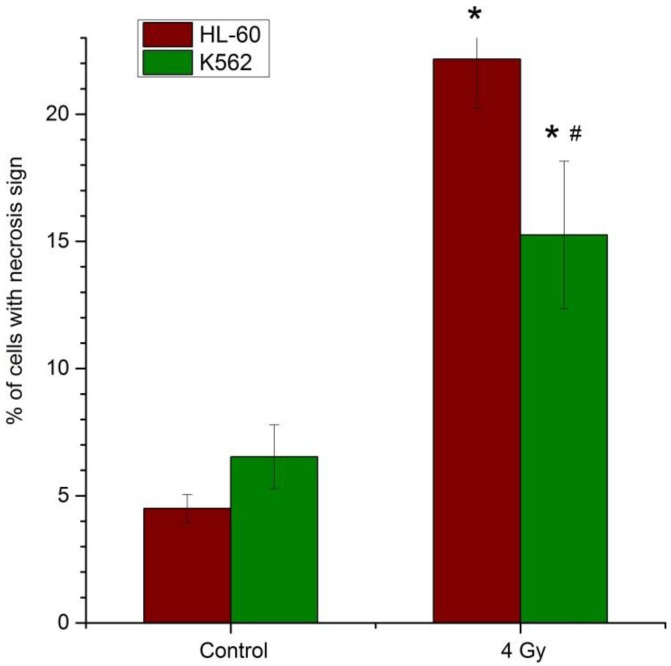
Percentage of cells with signs of necrosis in control group and after radiation exposure at the dose of 4 Gy in the HL-60 and K562 cell lines. *—Statistically significant difference between control and irradiated cells, *p* < 0.05; #—Statistically significant difference between the HL-60 and K562 cell lines, *p* < 0.05.

Figure 1 demonstrates the number of cells with the signs of necrosis in the HL-60 and K562 cancer cell lines after radiation exposure at the dose of 4 Gy. In the HL-60 cell line in the control group, the percentage of dead cells is 4.5%, and after exposure it increases up to 22%, which is about 5 times higher than in the control. In the radioresistant K562 cell line in the control group, the percentage of dead cells is 6% and after irradiation this value increases up to 15%.

### 2.2. Radiation-Induced Changes in microRNA Expression

**Figure 2:**
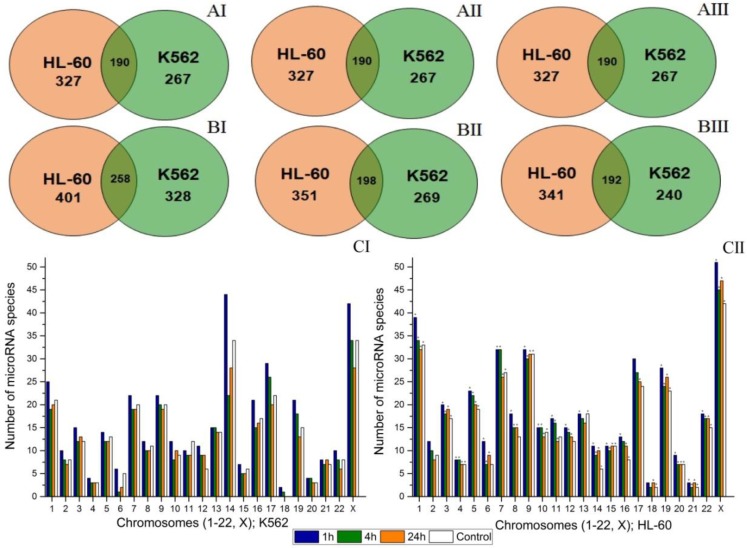
Effects of radiation exposure at the dose of 4 Gy on microRNA expression in the K562 and HL-60 cell lines. (**A**,**B**). The number of differentially expressed microRNAs in the HL-60 and K562 cell lines. **AI**, **AII**, **AIII**. The number of microRNAs in K562 and HL-60 cells in the control group 1, 4, and 24 h after irradiation, respectively. **BI**, **BII**, **BIII**. The number of microRNAs in K562 and HL-60 cells in the experimental group 1, 4, and 24 h after irradiation, respectively. **CI**. The number of expressed microRNAs depending on the chromosomal localization in the K562 cell line 1, 4, and 24 h after irradiation. **CII**. The number of expressed microRNAs depending on the chromosomal localization in the HL-60 cell line 1, 4, and 24 h after irradiation. *—Statistically significant difference from the K562 cell line, *p* < 0.05.

Figure 2A,B show the number of differentially expressed microRNAs in control and experimental groups in the HL-60 and K562 cell lines 1, 4, and 24 h after radiation exposure. Intersection of circles illustrates the number of the same microRNAs differentially expressed in both cell lines. The number of microRNAs in control groups is the same during the experiment (Figure 2AI–AIII). The most pronounced change in the number of expressed microRNAs occurs 1 h after irradiation (Figure 2BI). Compared to the control group, their number increases by 74 and 61 in the HL-60 and K562 cell lines, respectively. Four and twenty four hours after irradiation, the number of microRNAs in the experimental groups decreases gradually. For the experimental group of the K562 cell line, this value becomes lower than in the respective control group 24 h after irradiation (Figure 2BII,BIII). In the HL-60 and K562 cell lines, 258, 198, and 192, the same microRNAs specific for both cell lines, have been detected 1, 4, and 24 h after radiation exposure, respectively.

Figure 2C illustrates the data on the number of expressed microRNAs located in either chromosome at each point of the experiment. Figure 2C shows that for the K562 and HL-60 cell lines, the largest number of expressed microRNAs is located in chromosomes 14, X, and in chromosome X, respectively. In both cell lines, the lowest number of microRNAs is located in chromosome 18. The figure shows that the distribution of expressed microRNAs in chromosomes is different for two cell lines. In both cell lines, the number of expressed microRNAs varies during the experiment. The most considerable variations in the number of microRNAs in the K562 cell line has been observed for chromosomes 6, 12, 14, 17, 19, and X (Figure 2CI). In the HL-60 cell line, variations in the number of the expressed microRNAs are less pronounced, but still registered in chromosomes 1, 6, and X (Figure 2CII).

Thus, the most pronounced changes in microRNA expression profiles are recorded 1 h after irradiation. Changes in the number of expressed microRNAs have been observed for all chromosomes. Variations in the number of expressed microRNAs in chromosomes are more noticeable in the K562 cell line.

### 2.3. microRNA Effects on Signaling Pathway Activity

**Figure 3:**
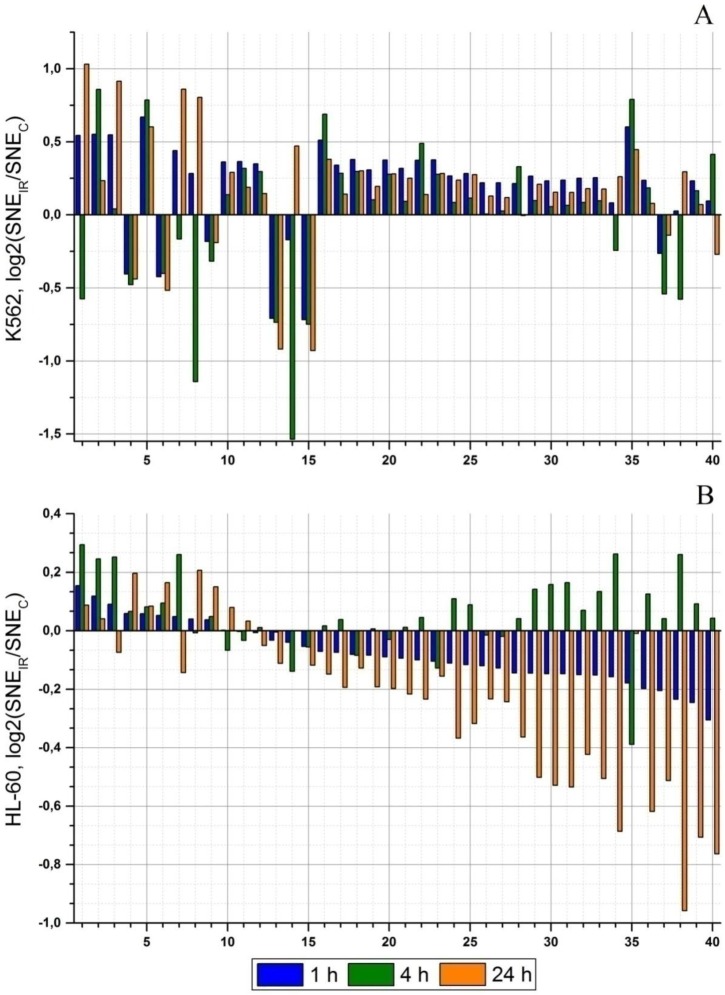
Dynamics of changes of microRNA effects on signaling pathways 1, 4, and 24 h after irradiation at the dose of 4 Gy. Only signaling pathways with statistically significant differences of log2(SNE_IR_/SNE_C_) values between the K562 and HL-60 cell lines are included at any experimental point (*p* < 0.01). (**A**) The K562 cell line. (**B**) The HL-60 cell line. Signaling pathway code: 1—Neuroactive ligand-receptor interaction (hsa04080); 2—Longevity regulating pathway—multiple species (hsa04213); 3—Ubiquitin mediated proteolysis (hsa04120); 4—Pertussis (hsa05133); 5—NF-kappa B signaling pathway (hsa04064); 6—Regulation of actin cytoskeleton (hsa04810); 7—Fanconi anemia pathway (hsa03460); 8—Amoebiasis (hsa05146); 9—PI3K-Akt signaling pathway (hsa04151); 10—Epstein-Barr virus infection (hsa05169); 11—AMPK signaling pathway (hsa04152); 12—Salmonella infection (hsa05132); 13—Shigellosis (hsa05131); 14—Type II diabetes mellitus (hsa04930); 15—Bacterial invasion of the epithelial cells (hsa05100); 16—Leukocyte transendothelial migration (hsa04670); 17—Bladder cancer (hsa05219); 18—Toll-like receptor signaling pathway (hsa04620); 19—Acute myeloid leukemia (hsa05221); 20—NOD-like receptor signaling pathway (hsa04621); 21—Hepatitis C (hsa05160); 22—HIF-1 signaling pathway (hsa04066); 23-RIG-I-like receptor signaling pathway (hsa04622); 24—ErbB signaling pathway (hsa04012); 25—Neurotrophin signaling pathway (hsa04722); 26—Oxytocin signaling pathway (hsa04921); 27—cGMP-PKG signaling pathway (hsa04022); 28—Rap1 signaling pathway (hsa04015); 29—Insulin signaling pathway (hsa04910); 30—Epithelial cell signaling in Helicobacter pylori infection (hsa05120); 31—GnRH signaling pathway (hsa04912); 32—Phospholipase D signaling pathway (hsa04072); 33—Choline metabolism in cancer (hsa05231); 34—VEGF signaling pathway (hsa04370); 35—Cell adhesion molecules (CAMs) (hsa04514); 36—Fc epsilon RI signaling pathway (hsa04664); 37—Melanogenesis (hsa04916); 38—Calcium signaling pathway (hsa04020); 39—Renal cell carcinoma (hsa05211); and 40—Long-term depression (hsa04730).

Regulation of gene expression by microRNA is a complex process. Note, one microRNA can regulate the expression of many genes and the expression of one gene can be regulated by many different microRNAs. So, the study of microRNA differential expression is uninformative for understanding of radioresistance mechanisms. Thus, for more comprehensive study of radioresisitance, we have analyzed the distribution of microRNA sum of normalized expression (SNE) for intracellular signaling pathways. In Figure 3, the diagrams demonstrate microRNA effect on particular signaling pathway activity. microRNA effect on the signaling pathways is described by the binary logarithm of the ratio of the sum of the normalized expressions (SNE) of the microRNA in the experiment to that in the control group: log2(SNE_IR_/SNE_C_). Negative values of log2(SNE_IR_/SNE_C_) show an increase of microRNA effect on a particular signaling pathway, i.e., signaling pathway activity decreases. Positive values of log2(SNE_IR_/SNE_C_) show a decrease of microRNA effect, so the signaling pathway activity increases. For convenience, each signaling pathway has been assigned with a code. Signaling pathways with the log2(SNE_IR_/SNE_C_) value higher or lower than 0.5 in at least one experimental point have been taken for analysis.

Figure 3 illustrates considerable difference in the effects from microRNAs on signaling pathways for the K562 and HL-60 cell lines. According to the type of dynamics of changes in microRNA effect on signaling pathways, they can be divided into three groups: (1) signaling pathways with the same dynamics of log2(SNE_IR_/SNE_C_) change; (2) signaling pathways with an opposite dynamics of log2(SNE_IR_/SNE_C_) change; (3) signaling pathways with a partially opposite dynamics of log2(SNE_IR_/SNE_C_) change.

The first group includes 4 signaling pathways (Figure 3, code 2, 5, 13, and 15). microRNA effect on Longevity regulating pathway—multiplespecies (hsa04213) (Figure 3, code 2) and NF-kappa B signaling pathway (hsa04064) (Figure 3, code 5)—has been decreased during the experiment. In the radioresistant K562 cell line the log2(SNE_IR_/SNE_C_) value is 2–3 times less than in the HL-60 cell line. On the contrary, Shigellosis (hsa05131) and Bacterial invasion of epithelial cell (hsa05100) signaling pathways (Figure 3, code 13, 15) demonstrate another tendency: microRNA effect on these signaling pathways is increased, and in the K562 cell line the log2(SNE_IR_/SNE_C_) value differs by 2–3 times compared to the HL-60 cell line.

The second group includes 12 signaling pathways (Figure 3, code 4, 6, 9, 18, 19–23, 26, 27, and 35). Pertussis (hsa05133), Regulation of actin cytoskeleton (hsa04810), and PI3K-Akt signaling pathway (hsa04151) (Figure 3, code 6, 9) in the HL-60 cell line have positive log2(SNE_IR_/SNE_C_) value, indicating the decrease of microRNA effect on these signaling pathways. In the K562 cell line, microRNA effect on these signaling pathways is increased. The rest of the signaling pathways (Figure 3, code 18, 19–23, 26, 27, 35) included in this group demonstrate an increase of microRNA effect in the HL-60 cell line and a decrease of microRNA effect in the K562 cell line. The largest difference in log2fc values between two cell lines is observed in Cell adhesion molecules (CAMs) (hsa04514) signaling pathway (Figure 3, code 35). The most significant difference is observed 4 h after irradiation: log2(SNE_IR_/SNE_C_) values are −0.39 and 0.78 in the HL-60 and K562 cell lines, respectively.

The third group (with a partially opposite dynamics of microRNA effect change) includes 24 signaling pathways (Figure 3, code 1, 3, 7, 8, 10–12, 14, 16, 17, 24, 25, 28–34, and 36–40). The most significant differences in the dynamics of microRNA effect changes are observed in 15 signaling pathways. In the K562 cell line, 4 h after irradiation, the log2(SNE_IR_/SNE_C_) values are −1.68 and −1.52 in Amoebiasis (hsa05146) (Figure 3, code 8) and Type II diabetes mellitus (hsa04930) (Figure 3, code 14) signaling pathways, respectively. In the HL-60 cell line, these values are insignificant. In 13 signaling pathways (Figure 3, code 28–40), considerable differences in the microRNA effect have been registered 24 h after radiation exposure. Twenty four hours after irradiation, microRNA effect value is sharply increased, ranging from −0.38 to −0.9 in the HL-60 cell line. In the K562 cell line, microRNA effect is insignificant but reduced compared to the control 24 h after irradiation.

Thus, analysis of the dynamics of microRNA effect on signaling pathways has revealed significant differences in 40 signaling pathways. After radiation exposure, the activity of these signaling pathways can differ significantly between the radioresistant K562 cell line and radiosensitive HL-60 cell line.

### 2.4. An Effect of Chromosome Abnormality on microRNA Expression

**Figure 4:**
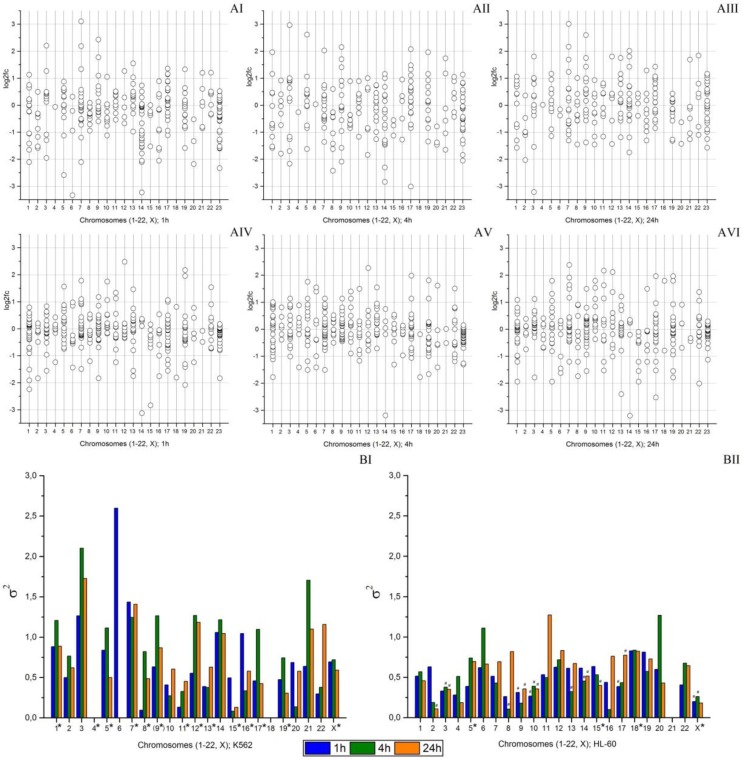
Analysis of microRNA expression dynamics depending on chromosome localization. (**A**) MicroRNA expression changes after radiation exposure at the dose of 4 Gy compared to control: **AI**—in the K562 cell line 1 h after irradiation; **AII**—in the K562 cell line 4 h after irradiation; **AIII**—in the K562 cell line 24 h after irradiation; **AIV**—in the HL-60 cell line 1 h after irradiation; **AV**—in the HL-60 cell line 4 h after irradiation; and **AVI**—in the HL-60 cell line 24 h after irradiation; (**B**) Dispersion of the log2fc values of microRNAs located in the chromosomes of the K562 and HL-60 cell lines: BI—the K562 cell line; BII—the HL-60 cell line. *—chromosomes with abnormalities (excluding translocations). (9*)—the ninth pair of chromosomes is absent in the K562 cell line but is part of the marker chromosomes. #—statistically significant difference from the K562 cell line, *p* < 0.05.

There are significant differences in the number of chromosomes between the HL-60 and K562 cell lines. The K562 cell line has monosomy or trisomy in 14 pairs of chromosomes, whereas the HL-60 cell line has only 4 pairs of chromosomes with such abnormalities. Changes in the amount of DNA in the cell can significantly affect gene expression. The diagrams in Figure 4A show variation of the log2fc values for all expressed microRNAs distributed according to their location in chromosomes in the HL-60 and K562 cell lines. At all points of the experiment, microRNAs in the K562 cell line have a greater scattering of log2fc values (Figure 4AI–AIII), indicating significant differences in microRNA expression compared to the control group after radiation exposure. In the HL-60 cell line, the variation of the log2fc values is less and most microRNAs are concentrated around zero region (Figure 4AIV–AVI).

The differences in scattering of the log2fc values are clearly shown in Figure 4B, where the dispersion of log2fc values of microRNAs located in each chromosome is presented. Since there are less than 5 microRNAs located in chromosomes 4 and 18 of the K562 cell line (Figure 4BI) and chromosome 21 of the HL-60 cell line (Figure 4BII), the dispersion of log2fc values of microRNAs with localization in these chromosomes has not been calculated. As Figure 4B shows, the dispersion of log2fc values for the K562 cell line is significantly higher than it is for the HL-60 cell line. The dynamics of changes in dispersion of log2fc also differs in two cell lines. In the K562 cell line, differences in log2fc dispersion between the points of the experiment can be considered significant for 15 pairs of chromosomes, whereas in the HL-60 cell line, significant changes in dispersion of log2fc are registered for 5 pairs of chromosomes.

Thus, analysis of the dynamics of microRNA expression depending on chromosomal localization has shown a greater scattering of log2fc values for each pair of chromosomes in the K562 cell line compared to the HL-60 cell line.

**Figure 5:**
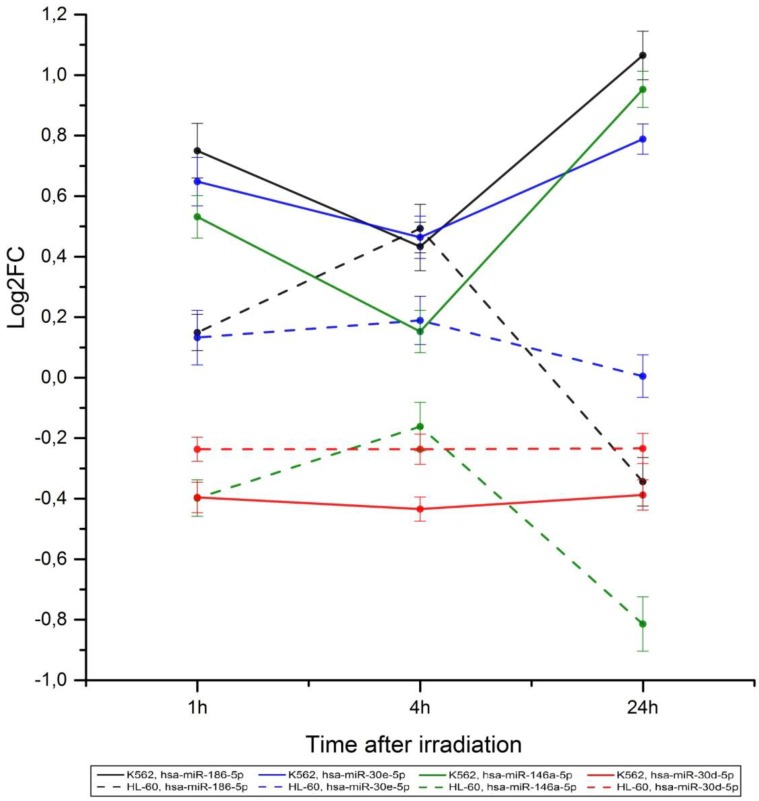
Comparative analysis of dynamics of the expression of microRNAs located in chromosomes with such abnormalities as trisomy and monosomy in the K562 and HL-60 cell lines.

One microRNA can regulate the expression of many genes and one gene can be a target for a large number of microRNAs. Table 1 lists the microRNAs participating in the post-transcriptional regulation of expression of genes included into signaling pathways with log2(SNE_IR_/SNE_C_) values less or more than 0.5 (Figure 3). Their chromosomal localizations are also listed in Table 1. As Table 1 shows, almost all microRNAs in the K562 cell line (except for hsa-miR-185-5p) are located in the regions that are a part of chromosomes with abnormalities. In the HL-60 cell line, only 5 microRNAs are located in the regions of chromosome abnormalities. Among the microRNAs presented in Table 1, there are 4 microRNAs located in chromosomes with simple abnormalities: hsa-miR-146a-5p (K562, −5,+5; HL-60, −5,−5), hsa-miR-186-5p (K562, −1,+1; HL-60, −1), hsa-miR-30e-5p (K562, −1,+1; HL-60, −1), and hsa-miR-30d-5p (K562, −8,+8; HL-60, −8). Figure 5 shows the dynamics of expression of these microRNAs after irradiation. The figure shows that, in general, the dynamics of change in microRNA expression after the radiation exposure correlates with the type of chromosome abnormality and the type of microRNA response to the stress. microRNA hsa-miR-186-5p and hsa-miR-30e-5p are located in chromosome 1. The K562 cell line has three chromosomes 1 [25], whereas the HL-60 cell line contains normal number of chromosome 1 [26]. In the K562 cell line, the expression of these microRNAs is higher than in the HL-60 cell line (Figure 5). A pronounced difference in the expression change is observed for microRNA hsa-miR-146a-5p located in chromosome 5. There are three chromosomes 5 and only one chromosome 5 in the K562 and HL-60 cell line, respectively (Table 1). The expression of microRNA hsa-miR-30d-5p (K562, −8, +8; HL-60, −8) decreases in response to radiation exposure, and the presence of an extra portion of microRNAs in the additional chromosome cannot greatly affect the changes in the expression profile. Figure 5 demonstrates small differences in the dynamics of microRNA hsa-miR-30d-5p expression between the cell lines.

Analysis of microRNA expression profiles in the K562 and HL-60 cell lines after radiation exposure has shown that chromosome abnormalities can change microRNA expression.

## 3. Discussion

For this study, we have selected two leukemia cell lines having different radiation sensitivity and different number of chromosomal abnormalities. The K562 cell line is less radiosensitive and has 15 chromosomal abnormalities. The HL-60 cell line possesses moderate radiosensitivity and 4 chromosomal abnormalities. The study of the dynamics of microRNA global expression has revealed that radiation exposure causes a significant change in the expression of microRNAs and, therefore, can have a significant effect on posttranscriptional regulation of gene expression. According to our experiments, more than half of all microRNAs in K562 and HL-60 cells change their expression after irradiation at the dose of 4 Gy (Figure 2A). These results are in agreement with the results of earlier studies reporting changes in global expression of microRNA in response to radiation exposure [27,28,29]. Most changes in the profiles of microRNA expression have been recorded 1 h after irradiation in both cell lines. The dynamics of the number of expressed microRNAs changing with the chromosomal location after irradiation show that changes in the amount of microRNAs are typical for all chromosomes and are more pronounced in the radioresistant K562 cell line.

The studies on distribution of genes encoding microRNAs in chromosomes in human assembly genome GRCh37 have shown that chromosomes 1, 2, 8, 11, 14, 17, 19, and X contain larger number of microRNA genes than other chromosomes. The least number of microRNA genes are located in chromosomes 13, 18, and 21. Chromosomes 1, 14, 19, and X are the major cancer-associated miRNA gene-containing chromosomes [30]. The performed analysis of chromosome localization of expressed microRNA in K562 and HL-60 cell lines has demonstrated that the distribution of these genes between chromosomes is similar to the human assembly genome GRCh37 (Figure 2). In the data obtained for human assembly genome GRCh37, the number of expressed microRNAs is always more than the number of microRNA genes located in all chromosomes in our experiment. In K562 cell line, the number of expressed microRNAs is less than in HL-60 cell line (Figure 2C). The amount of genetic material in K562 cell line is less than in HL-60 cell line. An increase of the amount of genetic material in K562 cell line is associated with a large number of marker and normal chromosomes. Additional genetic material produced from genomic mutations and chromosomal aberrations has a broken functional integrity leading to a decrease of its transcriptional activity [31,32].

Differences in response to the microRNA expression to irradiation can also indirectly indicate breaking of functional integrity of the genetic material. The values of the microRNA expression obtained for different chromosomes during the experiment with the radiation-resistant K562 line demonstrate greater scattering. Figure 4 shows that both the log2fc values and their dispersions have a wider spreading for the K562 cell line in comparison with HL-60.

Different signaling pathways play a significant role in formation of metastases, chemoresistance, and radiation resistance of cells. The identification of signaling pathways that potentially are responsible for radiation resistance of cancer cells is important for elaboration of appropriate methods of medical treatment and for development of new means of increasing radiation sensitivity of malignant tumors. Nowadays, many methods for evaluating the activation of signaling pathways are elaborated [33,34]. Commonly, these methods employ gene expression data and do not take into account the mechanisms of posttranscriptional regulation of gene expression by means of microRNAs, and also do not take into account the reasons causing changes in the expression of genes included into these signaling pathways [33].

The effect of microRNA on signaling pathways is evaluated as the binary logarithm of a ratio between the sum of the normalized microRNA expressions in irradiated cells and a similar sum in cells of the control group log2(SNE_IR_/SNE_C_). Our analysis reveals a difference in the microRNA pressure obtained for the radiation-resistant and radiation-sensitive cell lines. Fourty signaling pathways with high differences in log2(SNE_IR_/SNE_C_) have been revealed. Among them, one can see the signaling pathways already associated with radiation resistance (Figure 3, code 5, 7, 9, 16, 17, 18) [35,36,37,38]. However, there are lots of signaling pathways that have not been associated with the radiation resistance of cancer cells (Figure 3, code 1, 4, 10, 25, 30, 37, and 40). Moreover, they can hardly be associated with the mechanisms of radiation resistance of cancer cells. Nevertheless, our experiments have shown that log2(SNE_IR_/SNE_C_) values in radioresistant and radiosensitive cell lines are significantly different. We believe a large number of chromosome and genome mutations in the K562 cell line are responsible for these differences. Chromosome and genome mutations lead to change in the amount of genetic material and can mechanistically influence gene expression and signaling pathway function [22]. It has been demonstrated that changes in expression level in colorectal cancer cells correlate with alterations in DNA content, and large chromosomal segments containing multiple genes are transcriptionally affected in a coordinated way [32]. The effect of genome mutations on the messenger RNA expression has been demonstrated assuming its relation to the mechanistic processes of breaking genome integrity [39,40]. Studies on chromosomal aberrations and gene expression profiles in non-small cell lung cancer show that overexpressed genes are located in amplified regions while underexpressed genes are located in deleted regions [41]. Similar mechanisms are assumed to take place in the case of microRNA expression. For microRNAs, it has also been shown that abnormalities in expression of genes encoding microRNAs can result from chromosomal translocation leading to, in particular, overexpression of the gene encoding miR-125b, due to translocation of t(2;11) in some types of leukemia [24]. In our opinion, localization of microRNA genes in the regions of chromosome abnormalities can lead to disturbances in normal expression of these microRNAs and changes in post-transcriptional gene regulation, thereby affecting signaling pathway activity. Thus, localization of specific microRNAs in regions with genome and chromosome abnormalities is responsible for differences in microRNA effects on signaling pathways that have not been associated with radioresistance.

To verify this hypothesis, we have analyzed the composition of microRNAs contributing to regulation of these signaling pathways (Table 1). Analysis of microRNA localization has demonstrated that most of the microRNAs in the K562 cell line are located in abnormal chromosome regions. It confirms our assumption that differences in microRNA’s effect on signaling pathways cannot be associated with radioresistance and can be explained by localization of specific microRNAs in genome abnormality regions.

For further analysis, microRNAs located in chromosomes with monosomy and trisomy have been selected. Chromosomes with this type of mutation are not subjected to large-scale functional rearrangements and can preserve mechanisms for the regulation of gene expression typical for normal chromosomes. Commonly, the expression of genes located in chromosomes with trisomy is increased [42] and in chromosomes with monosomy is reduced [43]. In our experiments, 4 microRNAs (hsa-miR-30d-5p, -30e-5p, -146a-5p, -186-5p) have been located in chromosomes with trisomy and in normal chromosomes or in chromosomes with monosomy, and in the K562 and HL-60 cell lines, respectively. After irradiation, the expression of microRNAs located in chromosomes with trisomy occurs to be higher than the expression of microRNAs located in normal chromosomes or in chromosomes with monosomy (Figure 5). It is clearly seen in microRNA has-mir-146a-5p: the gene is located in chromosome 5. Trisomy 5 and monosomy 5 are typical for K562 and HL-60, respectively. microRNA hsa-mir-146a-5p can regulate a number of genes included, in particular, in signaling pathways Pertussis (hsa05133), Epstein-Barr virus infection (hsa05169), Hepatitis C (hsa05160), and long-term depression (hsa04730), with Fanconi anemia pathway (hsa03460) (Figure 3, code 4, 7, 10, 21) having significantly different log2(SNE_IR_/SNE_C_) values in radioresistant and radiosensitive cell lines. However, comparing hsa-mir-146a-5p gene localization in the K562 and HL-60 cell lines and its expression (Table 1, Figure 5), one can conclude that the observed differences in log2(SNE_IR_/SNE_C_) values are related to mechanistic effects of chromosome abnormalities and are specific for our cell lines only.

Thus, we can explain partially an absence of single-valued data on microRNAs, genes, and signaling pathways associated with radioresistance of cancer cells. If the location of chromosomes and the presence of genomic mutations and chromosomal aberrations are not taken into account in the analysis of microRNA expression, one can conclude that these microRNAs are markers of radioresistance in cancer cells. However, in reality, the difference in the microRNA expressions is due to chromosomal aberrations in the studied cell lines.

## 4. Materials and Methods

In the experiments we used:

(1) the HL-60 cell line, a radiosensitive human promyelocytic leukemia cells (karyotype 44,X,−Х,−5,dic(5;17)(q11;p11),del(7)(p?),der(7)t(5;7)(q11;q?31),t(5;16)(q11;q?),add(8)(q?),der(9)del(9)(p2?),t(9;14)(q2?;q2?),del(10)(p?),ins(11;8)(q13?;?),der(14)t(14;15)(q1?;q?),−15,der(16)t(5;16)(q?;q?22~24),der(16)t(7;16)(?;q?22~24),+18) [26];
(2) the К562 cell line, a radioresistant chronic myeloid leukemia cells (karyotype 67,X,−X,+1,der(2)add(2)(q33),+4,+5,der(?)t(5;6),dup(6)(p12~p22),−7,inv(7),del(7)(p15),der(7)readel(7),+8,−9,−9,dup(9)(q34),del(9)(p12),der(10)t(3;10),der(10,17)t(3;10;17),+11,der(?)t(6;11),−12,der(12)t(12;19),der(12)t(12;21),der(?)t(12;19),−13,der(13)t(9;13),+15,+16,−17,der(17)t(9;17)×2,−19,der(19)t(2;19),der(?)t(19;20),der(22)(q11.2),del(X)(p11)) [25].

Cells were grown at 37 °C in a humidified atmosphere of 5% CO_2_. Cells were maintained in RPMI-1640 culture medium containing 10% FBS (PAA Laboratories GmbH, Pasching, Austria) that was supplemented with 50 μg/mL gentamycin (PanEco, Moscow, Russia) and L-glutamine (PanEco, Moscow, Russia).

Cell lines were exposed to X-rays at a dose of 4 Gy (photon energy of 10 MeV) using the Elekta Synergy linear accelerator (Elekta, Stockholm, Sweden). Cells were irradiated for 55 s at room temperature. Cancer cells were in 6 well plates. Transportation of the plates with cell cultures to the site of irradiation and back was performed in a thermostated container at a temperature of 37 °C. All experiments with the cells were carried out during the logarithmic growth phase. Manipulation with the control group was similar to the experimental group, except for irradiation.

Cell viability was evaluated using a mixture of fluorescent dyes: Propidium iodide and acridine orange. Analysis of stained cells was carried out using fluorescence microscopy methods [44].

The total number of microRNA-containing RNA was isolated from the cells using Absolutely RNA miRNA kit (Agilent Technologies, Santa Clara, CA, USA) 1, 4, and 24 h after irradiation. Using the Agilent 2100 Bioanalyzer capillary electrophoresis device, the quality of the isolated RNA was evaluated by the ratio of 18S/28S RNA. For further studies, samples were taken with the RNA integrity number (RIN) > 8.0. Using the NEBNext Small RNA Library Prep Set (NEB, Hitchin, UK), cDNA libraries were prepared from microRNA. Purification of the libraries was performed by electrophoresis in 6% polyacrylamide gel. The cDNA regions corresponding to the microRNA between 145 and 160 bp were cut from the gel and isolated. In all the microRNA libraries obtained, concentration of cDNA was measured using a Qubitfluorometer (Invitrogen, Carlsbad, CA, USA). Equimolar amounts (2 nM) of sample were taken from each library and a pool of libraries was prepared, which was sequenced using the MiSeq System (Illumina, San Diego, CA, USA) high-throughput sequencing system employing a 150-bp single-ended reading kit.

After sequencing, FASTQ files were obtained. Data were processed using the GenXPro omiRas services. As a result, tables were created with data that included the microRNA name and the number of microRNA normalized for 10^5^ reads. During the data processing, the number of differentially expressed microRNAs and the number of the same differentially expressed microRNAs in two cell lines were calculated. Further, a search was carried out for genes, the expression of which was regulated by the microRNAs obtained during the sequencing. To continue the analysis, microRNAs were taken with normalized expression > 100 pieces. As a result, lists of genes were created, the expression of which regulates microRNA with normalized expression > 100 pieces, for each cell line 1, 4, and 24 h after irradiation. The expression of microRNA was determined by the log2 ratio of the sums of normalized microRNA expressions in the experiment to the control value: log2(SNE_IR_/SNE_C_).

The search for signaling pathways comprising these genes was performed in the KEGG database (http://www.genome.jp/kegg/). Since each microRNA can have an effect on different genes, the normalized expression of microRNA for each gene (NEG) was calculated by the formula

$${\mathrm{NEG}}_{i}=\frac{\mu_{i}}{\sum_{j=1}^{n}I\left({miRNA}_{i}\to {gen}_{j}\right)},$$

where $\mu_{i}$ is the normalized expression of *i*-microRNA for 10^5^ reads, I(A) is the indicator of event *A*

$$I\left(A\right)=\{\begin{array}{ll} 1, & if\;A\;is\;true,\\ 0, & if\;A\;is\;false. \end{array}$$

GenXPro omiRas services were employed to list genes whose expression was regulated with participation of any individual microRNA. Sum of normalized expressions of microRNA for a signaling pathway was calculated by the formula

$${\mathrm{SNE}}^{k}=\sum_{j}I\left({gene}_{j}\in {path}_{k}\right)\cdot \sum_{i}{\mathrm{NEG}}_{i}\cdot I\left({miRNA}_{i}\to {gene}_{j}\right).$$

The sums of normalized expression of the experiment (irradiated cells) and control (unirradiated cells) were compared and given as the log2(SNE_IR_/SNE_C_) (the binary logarithm of the SNE ratio between experiment and control). Signaling pathways demonstrating the log2(SNE_IR_/SNE_C_) > 0.5 and the log2(SNE_IR_/SNE_C_) < −0.5 in at least one experiment were selected. The obtained data are shown in diagrams.

The localization of microRNA in chromosomes was determined using the National Center for Biotechnology Information Search database (https://www.ncbi.nlm.nih.gov/).

Dispersion of the log2fc values for each individual pair of chromosomes (σ^2^) was calculated by the formula $\sigma^{2}=\frac{\sum_{i=1}^{n}{\left(x_{i}-\bar{x}\right)}^{2}}{\left(n-1\right)}$ (where $x_{i}$ is the log2fc value of particular microRNA located in this pair of chromosomes and $\bar{x}$ is the average value of the log2fc for all microRNAs expressed by this cell line). The dispersion was calculated only for chromosomes comprising more than 5 microRNAs.

All experiments were tripled and data were given as M ± SD (mean value ± standard deviation). Differences between irradiated and control cells are regarded as statistically significant when *p* calculated by the two-sided Student *t*-test is <0.05.

Cell cultures used in the research were obtained from RCCC of V (Russian cell culture collection of Vertebrates) (Saint Petersburg, Russia). No human/animal directly participated in biomaterial sampling process during this research. Local Russian regulations require no approval for using cell line biomaterials for scientific research purposes (Federal Law of June 23, 2016 No. 180-FZ).

## 5. Conclusions

An analysis of radiation-induced change in the profiles of the microRNA expression of the K562 and HL-60 cancer cell lines allows for the conclusion that the change in expression in response to the radiation exposure in our experiments can depend on the number of chromosome aberrations and the genome mutations of cell lines. microRNA localization and the presence of chromosome aberrations and genome mutations have to be taken into account in estimation of the relation between the radioresistance of cancer cells and profiles of microRNA expression.

## Figures and Tables

**Table 1:** Chromosome localization of microRNAs involved into post-transcriptional regulation of gene expression and included in signaling pathways with the log2(SNE_IR_/SNE_C_) values higher or lower than 0.5.

microRNA	Chromosome Localization		Number of Signaling Pathways
	K562 (67, X)	HL-60 (44, X)	
hsa-miR-101-3p	1,+1 (1p31.3); м7 (9p24.1)	1 (1p31.3); 9 (9p24.1)	2
hsa-miR-103a-3p	5,+5 (5q34); 20 (20p13)	5,−5 (5q34); 20 (20p13)	1
hsa-miR-16-5p	13,−13 (13q14.2); 3 (3q25.33); м15 (13q14.2)	13 (13q14.2); 3 (3q25.33)	29
hsa-miR-181a-5p	1,+1 (1q32.1); м19 (1q32.1); м8 (9q33.3)	1 (1q32.1); 9 (9q33.3)	5
hsa-miR-181b-5p	1,+1 (1q32.1); м19 (1q32.1); м8 (9q33.3)	1 (1q32.1); 9 (9q33.3)	3
hsa-miR-24-3p	19,−19 (19p13.12); м7(9q22.32); м8 (9q22.32); м17 (19p13.12); м18 (19p13.12)	9 (9q22.32); 19 (19p13.12)	22
hsa-miR-7-5p	15,+15 (15q26.1); 19,−19 (19p13.3); м7 (9q21.32); м8 (9q21.32)	9 (9q21.32); 15,−15 (15q26.1); 19 (19p13.3)	22
hsa-miR-92a-3p	13,−13 (13q31.3); м15 (13q31.3)	13 (13q31.3)	5
hsa-miR-9-5p	1,+1 (1q22); 5,+5 (5q14.3); 15,+15 (15q26.1)	1 (1q22); 5,−5 (5q14.3); 15,−15 (15q26.1)	22
hsa-miR-17-5p	13,−13 (13q31.3); м15 (13q31.3)	13 (13q31.3)	26
hsa-miR-20a-5p	13,−13 (13q31.3); м15 (13q31.3)	13 (13q31.3)	8
hsa-miR-21-5p	17,−17 (17q23.1); м16 (17q23.1), м16 (17q23.1)	17 (17q23.1)	3
hsa-miR-23a-3p	19,−19 (19p13.12); м17 (19p13.12); м18 (19p13.12)	19 (19p13.12)	3
hsa-miR-27a-3p	19,−19 (19p13.12); м17 (19p13.12); м18 (19p13.12)	19 (19p13.12)	5
hsa-miR-186-5p	1,+1 (1p31.1)	1 (1p31.1)	5
hsa-miR-30e-5p	1,+1 (1p34.2)	1 (1p34.2)	2
hsa-miR-99a-5p	21 (21q21.1); м13 (21q21.1); м19 (21q21.1)	21 (21q21.1)	8
hsa-miR-155-5p	21 (21q21.3); м13 (21q21.3); м19 (21q21.3)	21 (21q21.1)	33
hsa-miR-185-5p	22 (22q11.21)	22 (22q11.21)	18
hsa-miR-146a-5p	5,+5 (5q33.3)	5,−5 (5q33.3)	12
hsa-miR-106b-5p	7,−7 (7q22.1); м4 (7q22.1); м5 (7q22.1)	7 (7q22.1)	8
hsa-miR-93-5p	7,−7 (7q22.1); м4 (7q22.1); м5 (7q22.1)	7 (7q22.1)	1
hsa-miR-29a-3p	7,−7 (7q32.3); м4 (7q32.3); м5 (7q32.3)	7 (7q32.3)	28
hsa-miR-30d-5p	8,+8 (8q24.22)	8 (8q24.22)	7
hsa-miR-27b-3p	м7 (9q22.32), м8 (9q22.32)	9 (9q22.32)	1
hsa-miR-199b-5p	м7 (9q34.11); м8 (9q34.11)	9 (9q34.11)	2
hsa-miR-126-3p	м7 (9q34.3); м8 (9q34.3)	9 (9q34.3)	3
hsa-miR-221-3p	X,−X (Xp11.3)	X,−X (Xp11.3)	12
